# Reference-Free Assessment of Speech Intelligibility Using Bispectrum of an Auditory Neurogram

**DOI:** 10.1371/journal.pone.0150415

**Published:** 2016-03-11

**Authors:** Mohammad E. Hossain, Wissam A. Jassim, Muhammad S. A. Zilany

**Affiliations:** Department of Biomedical Engineering, Faculty of Engineering, University of Malaya, Kuala Lumpur, Malaysia; University of Texas Health Science Center at San Antonio, Research Imaging Institute, UNITED STATES

## Abstract

Sensorineural hearing loss occurs due to damage to the inner and outer hair cells of the peripheral auditory system. Hearing loss can cause decreases in audibility, dynamic range, frequency and temporal resolution of the auditory system, and all of these effects are known to affect speech intelligibility. In this study, a new reference-free speech intelligibility metric is proposed using 2-D neurograms constructed from the output of a computational model of the auditory periphery. The responses of the auditory-nerve fibers with a wide range of characteristic frequencies were simulated to construct neurograms. The features of the neurograms were extracted using third-order statistics referred to as bispectrum. The phase coupling of neurogram bispectrum provides a unique insight for the presence (or deficit) of supra-threshold nonlinearities beyond audibility for listeners with normal hearing (or hearing loss). The speech intelligibility scores predicted by the proposed method were compared to the behavioral scores for listeners with normal hearing and hearing loss both in quiet and under noisy background conditions. The results were also compared to the performance of some existing methods. The predicted results showed a good fit with a small error suggesting that the subjective scores can be estimated reliably using the proposed neural-response-based metric. The proposed metric also had a wide dynamic range, and the predicted scores were well-separated as a function of hearing loss. The proposed metric successfully captures the effects of hearing loss and supra-threshold nonlinearities on speech intelligibility. This metric could be applied to evaluate the performance of various speech-processing algorithms designed for hearing aids and cochlear implants.

## Introduction

Performing listening tests with real subjects is a complex and time consuming process. It is also often difficult to measure the benefit of hearing aids which are intended to improve speech intelligibility for people with hearing loss. Speech intelligibility is reduced not only by the hearing loss, but also by environmental noise, room reverberation, speed of the speech by a speaker, distance from the speaker, intrinsic distortions within the auditory system including decreased frequency and intensity resolution at high presentation levels, central auditory nervous system dysfunction, and the nonlinear distortions introduced by the hearing aids. The assessment of speech intelligibility under adverse conditions has led to the development of several measurements and prediction procedures. The subjective scores could be predicted by replacing the human listener with a reliable model of the auditory system. A model that can predict the speech intelligibility scores for a given people with hearing loss would be an important tool for hearing-aid fitting or development of hearing-aid algorithms.

Objective measurements of speech intelligibility are calculated from original and distorted acoustic signals using a number of mathematical formulas. The articulation index (AI) [1], speech intelligibility index (SII) [2], speech transmission index (STI) [3, 4], and their variant methods are the common examples of objective measures that are applied directly to the acoustic stimulus waveform. In addition, an objective measure was proposed based on the signal-to-noise ratio in the modulation domain [5]. Also, the short-time objective intelligibility measure (STOI) using the ideal time frequency segregation method [6] was developed to predict the intelligibility of noisy and time-frequency weighted noisy speech. In contrast, the spectro-temporal modulation index (STMI) [7], neural articulation index (NAI), mean structural similarity index (MSSIM) [8], and neurogram similarity index measure (NSIM) [9] aim to predict speech intelligibility using computational models of the peripheral and/or central auditory system. Typically, all of these metrics, based on the property of the acoustic signal as well as the responses of the computational model, were developed using the full-reference method in which the features or responses of an original speech signal are compared to the features or responses to a distorted speech signal. In the present study, a reference-free metric is proposed based on the extraction of third-order statistical features from the responses of a computational model of the auditory periphery, i.e., the responses to an original (clean) acoustic signal are not required to estimate the speech intelligibility score, and thus the metric will be useful in situations where the access to the clean signal is either limited or impossible. Previously, an adaptive reference-free metric was proposed using speech-to-reverberation modulation energy ratio (SRMR) for predicting the subjective quality of reverberant and de-reverberated (reduced reverberation by algorithms) speech [10].

The responses of a population of model auditory-nerve (AN) fibers can be represented as a “neurogram”, in which the spike counts for AN fibers tuned to different characteristic frequencies (CFs) are plotted as a function of time. The features are extracted from the neurogram to estimate the intelligibility score. For example, in STMI [7], the joint spectro-temporal modulation information is extracted from the simulated neural responses. Similarly, the MSSIM [8] measures the mean similarity index between the unimpaired (reference) and impaired images. In this study, the proposed metric uses the bispectrum [11, 12] as a feature extractor from the auditory neurograms. The bispectrum is a third-order spectral representation of moments and cumulants of the signal and has been widely used in signal processing [12]. Computationally, bispectrum is a 2-D Fourier transform of third-order cumulants or moments of the signal. It can detect the phase coupling between the frequency components in a signal and thus provides more information about the signal than the second-order statistics such as power spectrum and autocorrelation function. Phase coupling indicates both frequency and phase coupling, in which the third frequency peak and its phase are the sum of the first two frequency peaks and phases, respectively.

In general, the second-order statistical measures, such as the autocorrelation function and power spectrum, work well for signals having a Gaussian (normal) probability density function. For many decades, the estimation of the power spectrum of discrete-time deterministic or stochastic signals has been a primary tool for digital signal processing applications, including radar, sonar, communication, speech, biomedical, geophysical, and imaging systems [13–15]. In power spectrum estimation, the signal under consideration is processed as a superposition of statistically uncorrelated harmonic components [16]. The information included in the power spectrum is essentially that which is present in the autocorrelation sequence and is sufficient to describe a Gaussian signal completely. The power spectrum (autocorrelation) can only represent Gaussian and stationary signals. However, most biological signals are non-Gaussian and non-stationary in nature. There are situations in which quadratic nonlinearity results phase coupling between two frequency components of a process through a contribution to the power at a frequency equal to their sum. Such coupling affects the third moment sequence and thus the bispectrum can be used in detecting such nonlinear effects. The power spectrum cannot be used for this purpose, because it suppresses the phase relations. The application of higher-order statistics (HOS) includes 1D pattern recognition [17, 18], chaotic signal characterization [19], telecommunication [20], analysis of bio-signals such as the ECG [21] and EEG [22]. The motivation to use higher-order spectra in signal processing is to derive information due to deviations from Gaussian statistics (normality), to determine the phase of non-Gaussian parametric signals, and to detect and characterize nonlinear mechanisms which generate time series via the phase relations of their harmonic components [23].

In this study, the proposed method is motivated by the above-mentioned applications of HOS in the field of signal processing. Also, most of the existing acoustic signal property-based speech intelligibility metrics such as the AI, SII, STI, SRMR, and STOI use information mainly related to the second-order statistics of the signal. However, it is well-known that most auditory processing stages respond in a nonlinear way. As the auditory periphery is the sole conduit for acoustic information to reach the higher auditory centers, the inclusion of nonlinear transformation of acoustic signals in the model of the auditory periphery is necessary to determine how changes or deficits in certain underlying mechanisms in the healthy or impaired ear may affect perception. In this study, an auditory-nerve (AN) model developed by Zilany and colleagues [24, 25] has been employed to simulate the neural responses to speech signals. This model incorporates most of the nonlinearities observed at the level of the AN, such as nonlinear tuning, level-dependent phase, compression, suppression, shift in the best frequency as a function of level, adaptation, as well as some other nonlinearities seen at high sound pressure levels [26, 27]. The application of HOS to the simulated neural responses (with all nonlinearities) is expected to provide results that reflect the pattern of human behavioral performance (subjective scores) in response to acoustic stimuli. To our knowledge, there is no previous work that uses the bispectrum as a feature extractor from auditory neurograms. The proposed metric is used to predict speech intelligibility for normal-hearing listeners under diverse background conditions. As the AN model used in this study can successfully simulate neural responses for listeners with hearing loss [28, 29], the metric is extended to predict intelligibility for people with different degrees of hearing loss.

When a complex broadband sound is analyzed in the cochlea of a normal ear, the result is a series of bandpass-filtered signals, each corresponding to one position on the basilar membrane. Each of the band-passed signals can be considered as a slowly varying envelope (ENV) superimposed on a more rapid temporal fine structure (TFS). Both ENV and TFS information are represented in the timing of neural discharges, although TFS information depends on phase locking to individual cycles of the stimulus waveform [30]. Thus, two types of neurograms were considered in this study. It has been reported in the literature that ENV is responsible for speech perception, whereas TFS is associated with melody and pitch perception as well as sound localization [31]. The features of the bispectrum are computed from the ENV and TFS neurograms in order to determine an objective measure of speech intelligibility.

## Methods

The following section describes the computational procedure of the proposed method. In addition, a brief description of the computational model of the auditory periphery is provided.

### Model of the Auditory System

Zilany and colleagues developed a computational model of the auditory periphery, and the responses of the model were validated against a wide range of physiological responses of AN fibers from the literature [25]. The AN model consists of several stages, each providing a phenomenological description of a major functional block in the auditory periphery, from the middle ear to the AN fiber. The schematic diagram of the AN model is given in Fig 1 of Zilany et al. [28]. The input to the model is an instantaneous pressure waveform of the stimulus which is passed to the middle-ear filter (ME). The output of ME filter is passed to a basilar membrane (BM) filter. The feed-forward control path controls the gain and bandwidth of the basilar membrane (BM) filter to account for several level-dependent properties in the cochlea. The signal is then passed through a model of the inner-hair-cell (IHC), which transduces the mechanical responses of the BM to an electrical potential. The output of the IHC low-pass filter is passed to the IHC-AN synapse model which determines the spontaneous rate, adaptation properties, and rate-level behavior of the AN model. Finally the discharge (spike) times are produced by a non-homogenous Poisson process that includes refractory effects.

**Fig 1:**
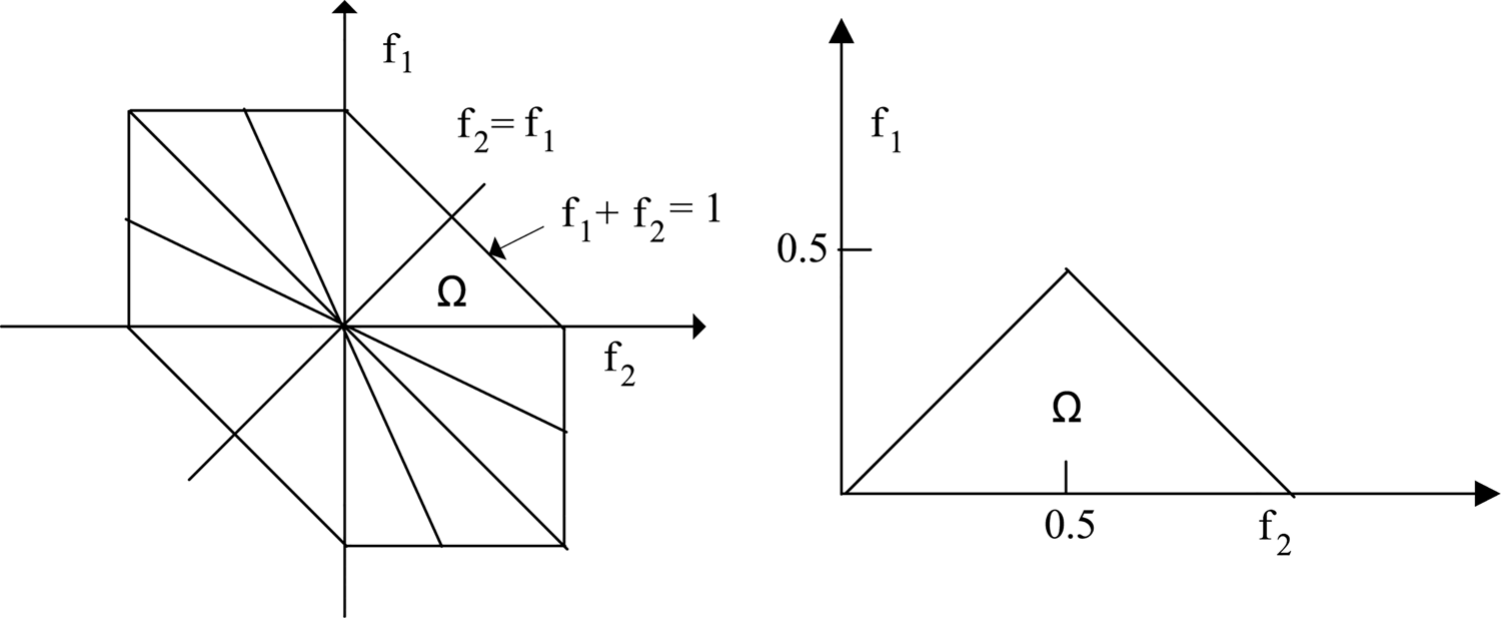
Non-redundant area of computation of the bispectrum for real signals. The features of the bispectrum are calculated within the triangular area Ω.

It has been reported in the literature that the IHC and outer-hair-cell (OHC) stereocilia are damaged by noised-induced impairment in the cochlea [32–34]. The damage in the OHC stereocilia causes both elevated threshold and broadened tuning of the AN-fibers, whereas damage in the IHC stereocilia results in only an elevation of the tuning curve without any substantial broadening of the tuning. The nonlinear phenomena observed in the cochlea such as the compressive changes in gain and bandwidth as a function of stimulus level, the associated changes in the phase of phase-locked responses, and two-tone suppression have all been related to a single mechanism in the inner ear, referred to as the cochlear amplifier [26, 27]. It has been reported that the damage to the OHC is also related to the reduction in the compressive nonlinearities in the BM responses [35] as well as to the reduction in the two-tone suppression of the auditory-nerve fiber responses [36]. Thus the outer hair cells perform an amplifying role, and it is the inner hair cells that detect the sound and transmit it to the brain via the auditory nerve. The IHC and OHC status are incorporated in the model by introducing scaling factors, 0 ≤ C_IHC_ ≤ 1 affects IHC transduction function and 0 ≤ C_OHC_ ≤ 1 affects the control path output [28, 29, 37].

The tuning characteristics of an auditory-nerve fiber at threshold are represented by the frequency threshold curve. The tuning parameters of the model AN fibers (Zilany and Bruce, 2006) are initially adjusted based on the Q10 values recorded from the cat AN fibers [38]. In light of the current debate on human cochlear tuning [39, 40], some parameters of the updated version of this model (employed in this study) are modified to better match human anatomy and physiology. These modifications include the middle-ear filtering, the cochlear place-frequency map, and the sharpness of cochlear frequency tuning [41].

### Neurogram

A neurogram is a two-dimensional representation in which neural responses of a wide range of characteristic frequencies (CFs) are displayed as a function of time. In this work, neurograms were created by simulating the responses of 32 AN fibers with CFs logarithmically spaced from 250 to 8000 Hz. The responses of three types (high, medium, and low spontaneous rates) of AN fibers were simulated. Final responses were weighted by the reported distribution of spontaneous rates (SR) of AN fibers (60% high, 20% medium, and 20% low) [33]. Based on resolution, two neurogram representations were created by averaging the neural responses of each CF with a bin size of 10 μs for TFS and 100 μs for ENV. Then the neural responses of each CF were divided into frames using a Hamming window (50% overlap) of 32 samples for TFS and 128 samples for ENV [42]. The combination of binning to 10 μs and smoothing with the 32-sample Hamming window accounted for spike synchronization to frequencies up to ~6.25 kHz, whereas the binning to 100 μs and smoothing with a 128-sample Hamming window resulted in synchronization frequencies that were limited to ~160 Hz. Thus the ENV neurogram excludes spike timing information about the temporal fine structure, but the TFS neurogram includes it. However, it is to be noted that when acoustic signals are passed through cochlear filters to get the corresponding neural responses, there is not a one-to-one mapping between acoustic and neural ENV or TFS information [43].

### Higher Order Statistics: Bispectrum

The HOS are spectral representations of moments and cumulants that are defined for deterministic signals and random processes. In this study, the features related to the third order statistics of the signal, referred to as the bispectrum, were considered. The bispectrum is the Fourier transform of the third order cumulant sequence of a stationary random process and is given by [11, 12]

$$\text{B }\left(f_{1},\;f_{2}\;\right)=\text{E }\left[\text{X}\left(f_{1}\right)\text{ X}\left(f_{2}\right)\;X^{*}\left(\;f_{1}\;+\;f_{2}\right)\right]$$ (1)

where X(*f*) is the Fourier transform of the random signal x(nT), n is an integer index, T is the sampling interval, E[.] stands for the expectation operation, and * represents the complex conjugate.

The bispectrum is a complex-valued function of two frequencies (*f*_*1*_, *f*_*2*_). The frequency is normalized by the Nyquist frequency to be between 0 and 1. The range of frequency indicates the extent of phase coupling between frequency components [23]. Generally, phase coupling occurs because of the nonlinear interaction between harmonic components. The signal consisting of three sinusoids [with frequencies and phases (*f*_*1*_, *ϕ*_*1*_), (*f*_*2*_, *ϕ*_*2*_) and (*f*_*3*_, *ϕ*_*3*_)] is said to be quadratic phase coupled if and only if *f*_*3*_ *= f*_*1*_ *+ f*_*2*_ and *ϕ*_*3*_ *= ϕ*_*1*_ *+ ϕ*_*2*_. The phase relations are easily investigated by the quadratic phase coupling. The power spectrum cannot explain the phase relations between harmonic components whereas the bispectrum is suitable for detecting the phase relations in many applications [44].

B(*f*_*1*_, *f*_*2*_) is a symmetric function. For real processes, there are 12 symmetry areas in the bispectrum. The bispectrum in the triangular area (Ω) is calculated by the conditions, *f*_*2*_ *≥ 0*, *f*_*1*_ *≥ f*_*2*_, and *f*_*1*_ *+ f*_*2*_ ≤ *1*. The triangular area can describe the whole bispectrum as shown in Fig 1. The features are calculated within the triangular area (Ω).

In this study, the features of the bispectrum were used to develop the proposed metric. It has been reported in the literature that different types of features can be extracted from bispectrum. These features include mean magnitude (*M*_*ave*_) and phase entropy (*p*_*e*_) [45], the weighted center of bispectrum (*f*_*1m*_, *f*_*2m*_) [46], and the normalized bispectral entropy (*p*_*1*_, *p*_*2*_) [11]. Entropy is used to characterize the regularity or irregularity of the signal and is defined as the expected value (i.e. the average amount) of the information from the bispectrum plot. Also, several other features related to the moment were considered to extract information from the bispectrum [47] such as the sum of logarithmic amplitudes (*H*_*1*_), the sum of logarithmic amplitudes of diagonal elements (*H*_*2*_), and the first-order spectral moment of amplitudes of diagonal elements in the bispectrum (*H*_*3*_). All the features were calculated in this work. However, the magnitude of some features did not change consistently as a function of hearing loss. This study only considered the *H*_1_, *H*_*2*_, and *H*_*3*_ features, because the magnitude of these features either increased or decreased consistently as a function of the degree of hearing loss.

The features *H*_*1*_, *H*_*2*_, and *H*_*3*_ are defied as:

$$H_{1}=\sum_{\Omega }\text{log}\left(\left|\text{B }\left(f_{1},\;f_{2}\right)\right|\right)$$ (2)

$$H_{2}=\sum_{\Omega }\text{log}\left(\left|\text{B }\left(f_{k},\;\;f_{k}\right)\right|\right)$$ (3)

$$H_{3}=\;\sum_{k=1}^{N}\;k\;\text{log}\left(\left|\text{B }\left(f_{k},\;\;f_{k}\right)\right|\right)$$ (4)

### Test Corpora

Two standard speech databases, Texas Instrument and Massachusetts Institute of Technology (TIMIT) [48] and Northwestern University Auditory Test No. 6 (NU6) [49]), were used in this study to evaluate the performance of the proposed method. It is to be noted that no behavioral study using human subjects was performed as part of the present study, rather the subjective scores collected by Studebaker et al. [50] were used to validate the proposed metric. Speech intelligibility was predicted for listeners with different degrees of hearing loss using a wide range of phoneme utterances from TIMIT [48]. TIMIT is a corpus of phonemically and lexically transcribed speech of American English speakers of different sexes and dialects. The speech signal of TIMIT is sampled at 16 kHz. The TIMIT data set consists of fifty eight different types of phonemes and six phonemes groups. The total phoneme utterance in the core test set is 7753. All of these phonemes were used in this study.

The NU6 [49] database was used to validate the proposed metric by comparing the performance of the proposed method to the subjective scores from behavioral studies by Studebaker et al. [50]. NU6 is a corpus of 200 monosyllabic words from a male speaker recorded by Auditec of St. Louis, sampled at 44.1 kHz.

### Procedure

**Fig 2:**
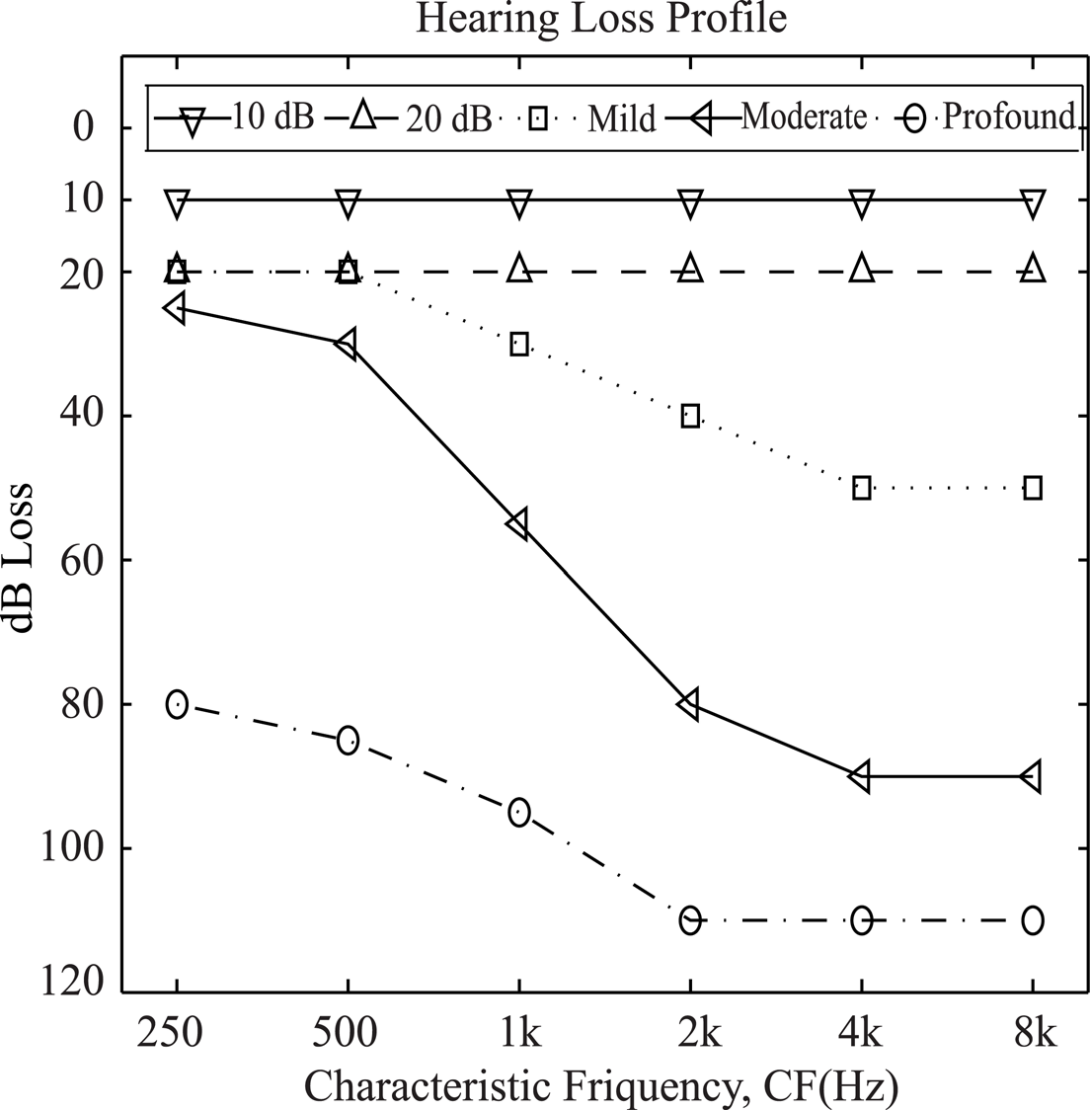
Profiles of hearing loss.

**Fig 3:**
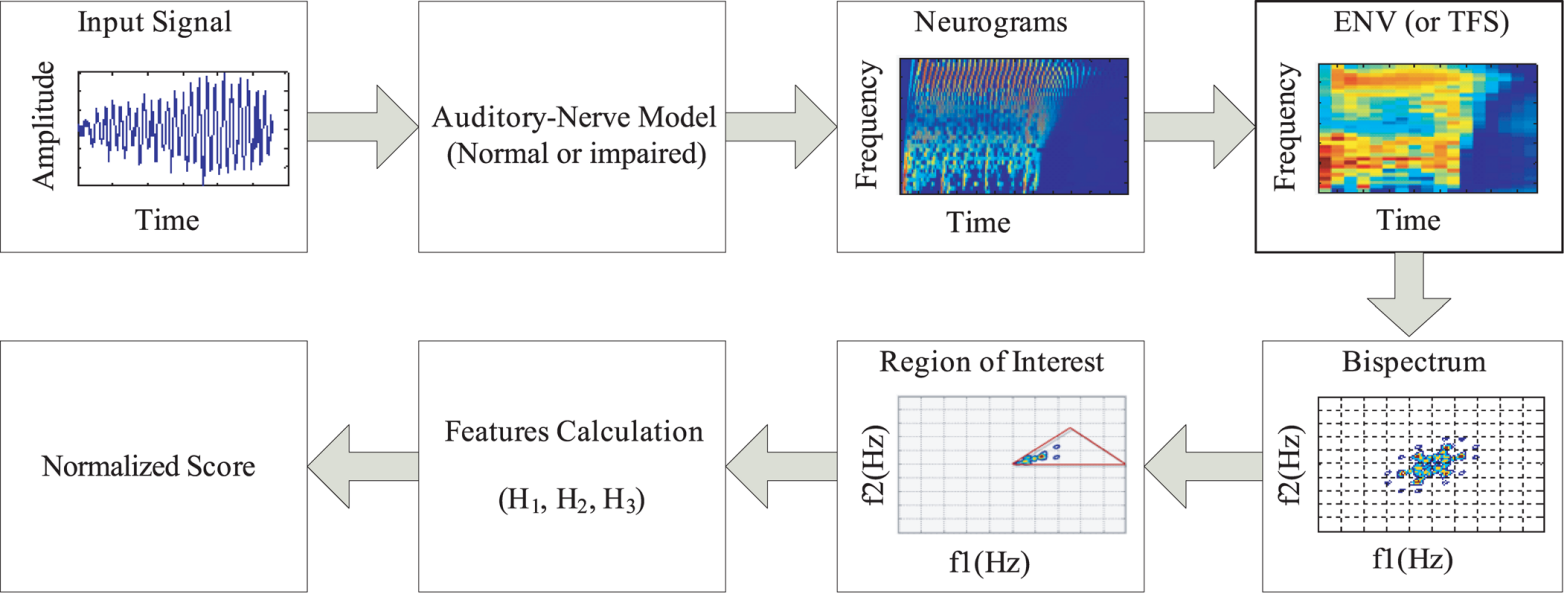
Block diagram of the proposed method. The speech signal was applied as an input to the model of the auditory-nerve (AN) fibers, and responses of the normal or impaired auditory systems were simulated for a wide range of characteristic frequencies to construct neurograms. The neurogram responses were smoothed to reflect envelope information (ENV) or all information temporal fine structure (TFS), and the bispectrum was estimated from the ENV or TFS neurogram. Finally the features were computed from the triangular area and normalized in order to estimate speech intelligibility score.

The input to the AN model was the speech signal from TIMIT or NU6. The speech token was resampled at 100 kHz, as required by the AN model [25]. In this study, five different types of hearing loss profiles such as flat10, flat20, mild, moderate, and profound hearing loss were considered and are shown in Fig 2. The audiograms were taken from Dillon [51] to illustrate the typical hearing losses at six different frequencies, and thus the threshold shifts at other CFs were estimated by interpolating between the values at two adjacent frequencies. It has been reported that for an average listener, approximately two-thirds of the threshold shift measured in the audiogram can be attributed to OHC impairment and one-third to IHC impairment [52]. Accordingly, to simulate the responses of impaired AN fibers, the model parameters for inner hair cell (C_IHC_) and outer hair cell (C_OHC_) were adjusted to account for one-third and two-third of the threshold shift, respectively, for each CF. Both parameters were set to 1 for normal hearing and to 0 for complete impairment. The ENV and TFS neurograms were constructed using the output of the synapse model. The bispectrum was then estimated from the neurograms to predict intelligibility scores. The complete procedure of the proposed method is illustrated in Fig 3.

To estimate bispectrum, the direct FFT-based method implemented in the higher order spectral analysis (HOSA) Matlab toolbox [53] was employed in this study. In the direct method, the bispectrum was estimated as an average biperiodogram using Eq 1 for the neural responses of each CF. The number of fast Fourier transform (FFT) point was set to 256 points for ENV and 512 points for TFS without overlap. In this case, the size of the bispectrum array for each CF of ENV is 256 × 256 whereas the corresponding bispectrum array size for each CF of TFS is 512× 512. A Rao-Gabr window [23] with a size of 3 × 3 was used for the frequency-domain smoothing. Different features were calculated within the triangular area Ω: *M*_*ave*_, *f*_*1m*_, *f*_*2m*_, *p*_*1*_, *p*_*2*_, *H*_*1*_, *H*_*2*_, and *H*_*3*_ [11, 45–47] of the bispectrum estimated for each of the CF responses in the neurogram. Thus for each neurogram, 32 values (each neurogram was constructed using the neural responses of 32 CFs) of each feature were computed and averaged across them to provide an overall feature value.

**Fig 4:**
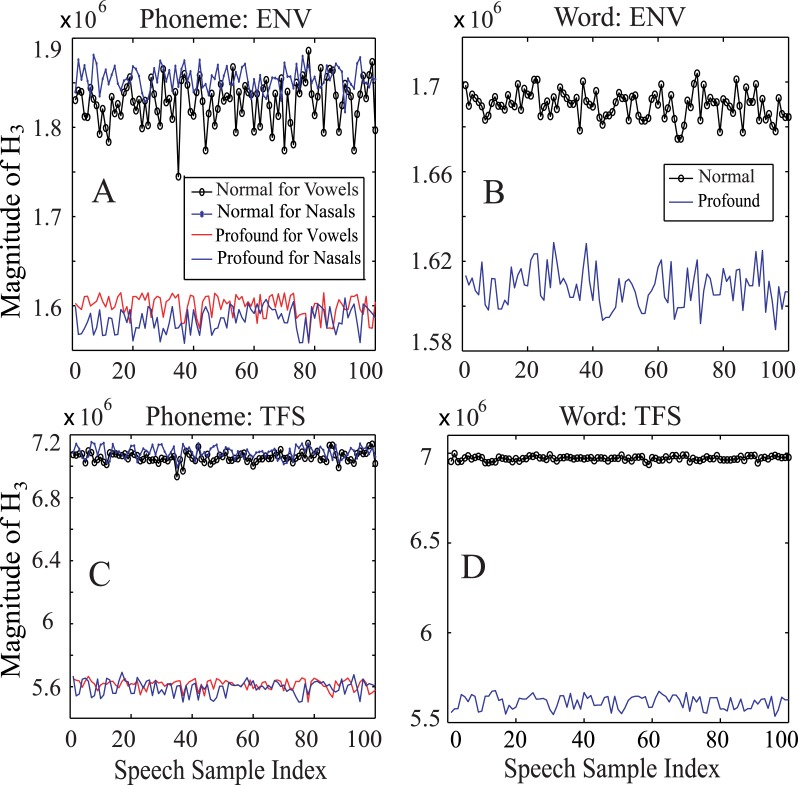
The magnitudes of *H*_*3*_ for normal-hearing and profound hearing loss are shown in response to 100 samples of phonemes (nasals and vowels) and words in quiet. (A, C): ENV and TFS responses for phonemes; (B, D): ENV and TFS responses for NU6 words.

It was found that the magnitude of three features (*H*_*1*_, *H*_*2*_, and *H*_*3*_) in the bispectrum was relatively constant for two extreme conditions (normal-hearing and profound hearing loss) irrespective of the input speech token, as shown in Fig 4. For the rest of the features, the magnitude was either not consistent as a function of hearing loss or not constant for two extreme conditions in response to phonemes and words, and thus they were not considered in this study to estimate speech intelligibility scores.

Fig 4 shows the dynamic range of *H*_*3*_ magnitudes calculated from the bispectrum of the phoneme neurograms (panels A and C) for each of two phoneme groups (nasal and vowel) as well as from the neurograms of words (panels B and D). The results are shown for 100 samples (randomly chosen) in all cases. The upper panels (A, B) show the result for ENV neurograms, and the lower panels (C, D) represent the responses from the TFS neurogram. The results are shown for speech signals presented at a conversational speech level of 65 dB SPL.

It was observed that the magnitude of *H*_*3*_ for profound hearing loss (in quiet) was comparable to the values estimated under noisy condition (at an SNR of -100 dB) for listeners with normal hearing, which allowed the same normalization to be applied to estimate speech intelligibility under noisy conditions. Also the magnitude of the bispectrum features consistently changed for people with hearing loss under noisy conditions. Thus, the behavioral results were compared to the predicted scores in quiet and under noisy conditions for listeners with and without hearing loss.

To obtain speech intelligibility scores between 0 and 1, the magnitude of a feature was normalized by the corresponding feature values for two extreme conditions (normal hearing and profound hearing loss). The score was computed using the following equation:

$$\text{Normalized Score}=\text{max}\left(\text{min}\left(\frac{Y\;-\;Y_{min}}{Y_{max}\;-\;Y_{min}},1\right),\;0\right)$$ (5)

where Y is the magnitude of the feature in the bispectrum of responses for listeners with a given hearing loss profile, and *Y*_*max*_ and *Y*_*min*_ are the values of the feature in the bispectrum of response for listeners with normal-hearing and profound hearing loss, respectively. The magnitude of *Y*_*max*_ and *Y*_*min*_ (averaged across all samples) for the features (*H*_*1*_, *H*_*2*_, and *H*_*3*_) is provided in Table 1 for different conditions (TFS vs. ENV neurogram, and phoneme vs. word).

**Table 1:** The reference values of three features (*H*_*1*_, *H*_*2*_, and *H*_*3*_) for ENV and TFS. Two types of signals were considered, phonemes and words.

Feature	ENV		TFS	
	Phoneme	Word	Phoneme	Word
***H***_***1***_: Y_max_(×10^5^)	2.02	1.77	7.25	7.14
***H***_***1***_: Y_min_(×10^5^)	1.69	1.48	5.19	5.22
***H***_***2***_: Y_max_(×10^3^)	3.12	2.90	5.90	5.77
***H***_***2***_: Y_min_(×10^3^)	2.60	2.50	4.44	4.48
***H***_***3***_: Y_max_(×10^6^)	1.84	1.69	7.05	6.97
***H***_***3***_: Y_min_(×10^6^)	1.59	1.61	5.58	5.61

## Simulation Results

This section describes the behavior of different bispectrum features as a function of hearing loss. In addition, the performance of the proposed method is evaluated and compared to the subjective scores from behavioral studies as a validation of the proposed metric.

### Intelligibility Scores for Different Phoneme Groups

**Fig 5:**
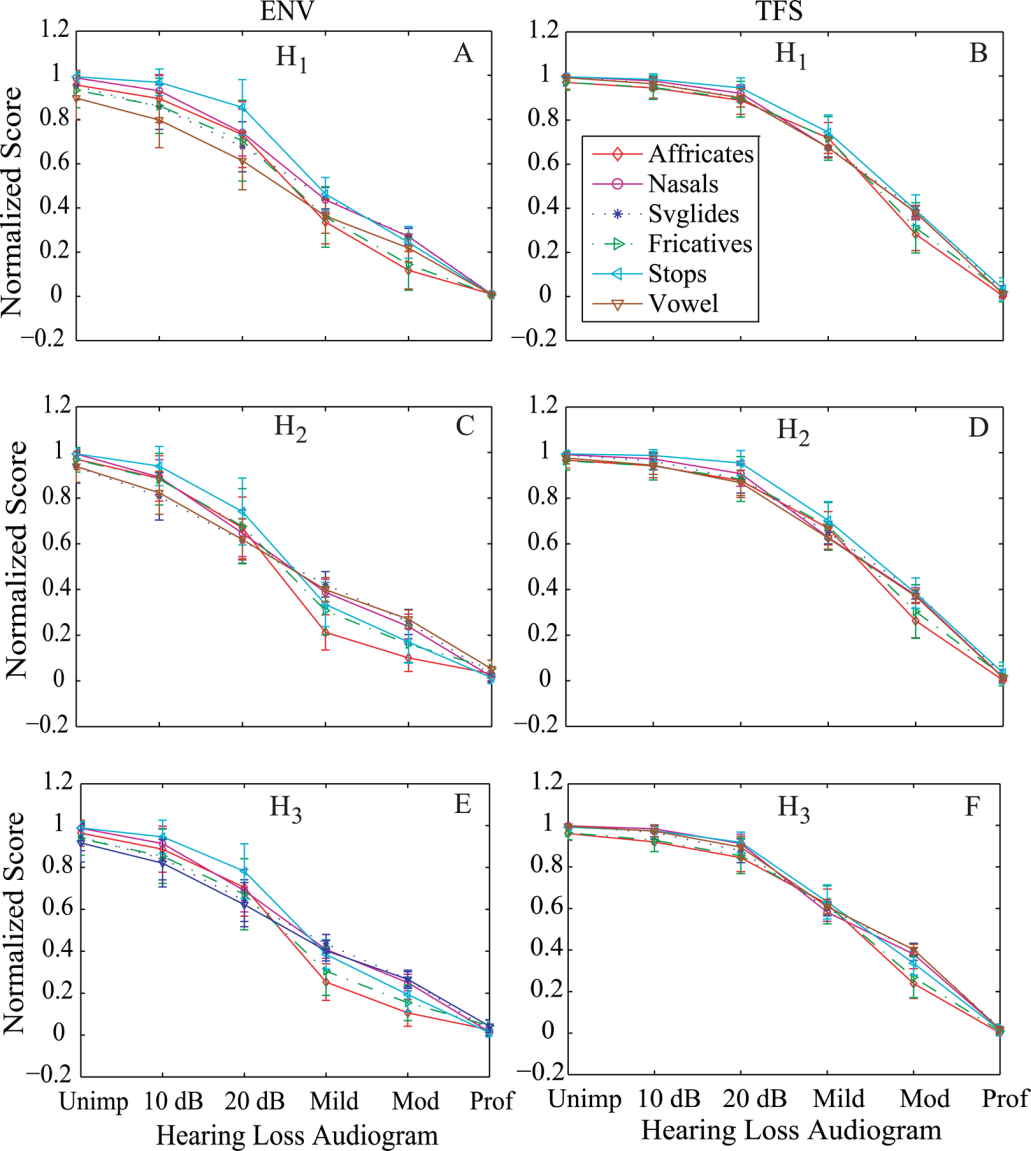
Normalized scores as a function of hearing loss for all phoneme groups from TIMIT database. Neurograms were constructed for the signals presented at 65 dB SPL, and the features (*H*_*1*_, *H*_*2*_, and *H*_*3*_) were computed from the ENV and TFS bispectrum responses. (A, C, E): Normalized score using ENV responses. (B, D, F): Normalized score using TFS responses.

Fig 5 shows the normalized predicted scores as a function of hearing loss using the features (*H*_*1*_, *H*_*2*_, and *H*_*3*_) computed from the bispectrum of neural responses for different groups of phonemes. The phonemes were taken from TIMIT database, and the responses are shown for six phoneme groups (fricatives, affricates, nasals, svglides, stops, and vowels). The phonemes were presented at 65 dB SPL. The normalized scores (Eq 5) are shown for ENV responses in the left panels (A, C, E), and the right panels (B, D, F) show the predicted score from the bispectrum of TFS responses. The score at any point represents the mean value of normalized scores for a listener with a particular profile of hearing loss in response to a phoneme group, and the error bars show the standard deviation of the scores.

It is clear that the scores for both ENV and TFS responses consistently declined for all phoneme groups as a function of the degree of hearing loss. Although the dynamic range of predicted scores was approximately same for both ENV and TFS responses, the normalized score from the ENV bispectrum showed a gradual roll-off with varying degree of hearing loss. On the other hand, the magnitude of normalized features (*H*_*1*_, *H*_*2*_, and *H*_*3*_) from the TFS bispectrum did not show any substantial change from completely unimpaired to mild hearing loss. This effect can be explained primarily by audibility, because ENV cues are more prominent in higher CFs [43], and there was significant high frequency hearing loss (40–50 dB) even in the mild case (Fig 2). In contrast, TFS cues are more prominent at lower frequencies [43], where the significant hearing loss was not shown until the moderate or profound cases (Fig 2).

In general, the predicted scores for fricatives and affricates dropped more quickly as a function of hearing loss compared to the scores for other phoneme groups. A similar observation was reported in [54] that the recognition performance of fricatives for listeners with steeply sloping hearing loss is lower compared to the scores for those with gradual or flat hearing loss.

Overall, it is clear that the normalized magnitude of features (*H*_*1*_, *H*_*2*_, and *H*_*3*_) for both ENV and TFS responses changed as a function of hearing loss, which enables the proposed metric to predict speech intelligibility as a function of hearing loss. If hearing thresholds of the listeners are within 10–15 dB of the normal hearing, most behavioral studies treat them as normal listeners [55, 56], and thus the recognition score for listeners with flat 10 dB hearing loss would be very close to that of normal hearing (very slower roll-off). On the other hand, the recognition score for listeners with profound hearing loss (more than 70–90 dB hearing loss in octave frequencies ranging from 250 Hz to 8 kHz) would be very low at a conversational speech level (~65 dB SPL), because the signal would be inaudible for most frequencies. The recognition score for listeners with mild and moderate hearing loss is expected to be in between the values for these two extreme conditions [54, 57]. Thus, it is required that the objective score from a metric should gradually decline or increase as a function of hearing loss. In general, the magnitude of three features (*H*_*1*_, *H*_*2*_, and *H*_*3*_) declined consistently as a function of the degree of hearing loss, and the results were comparable to each other. In this study, the feature *H*_*3*_ estimated from the ENV bispectrum was chosen for all subsequent analysis, because the result using *H*_*3*_ reflected the representative behavior of normalized scores for all three features, and the scores from the ENV responses declined more gradually than the scores from the TFS responses as a function of the degree of hearing loss. To test the statistical significance of predicted scores, the level of significance (*p*) was calculated by using a pair wise (two adjacent hearing loss-profile) t-test for all phoneme groups. The level of significance was found to be less than 0.01 (*p*<0.01) for all cases.

### Effects of SPL on Predicted Score

**Fig 6:**
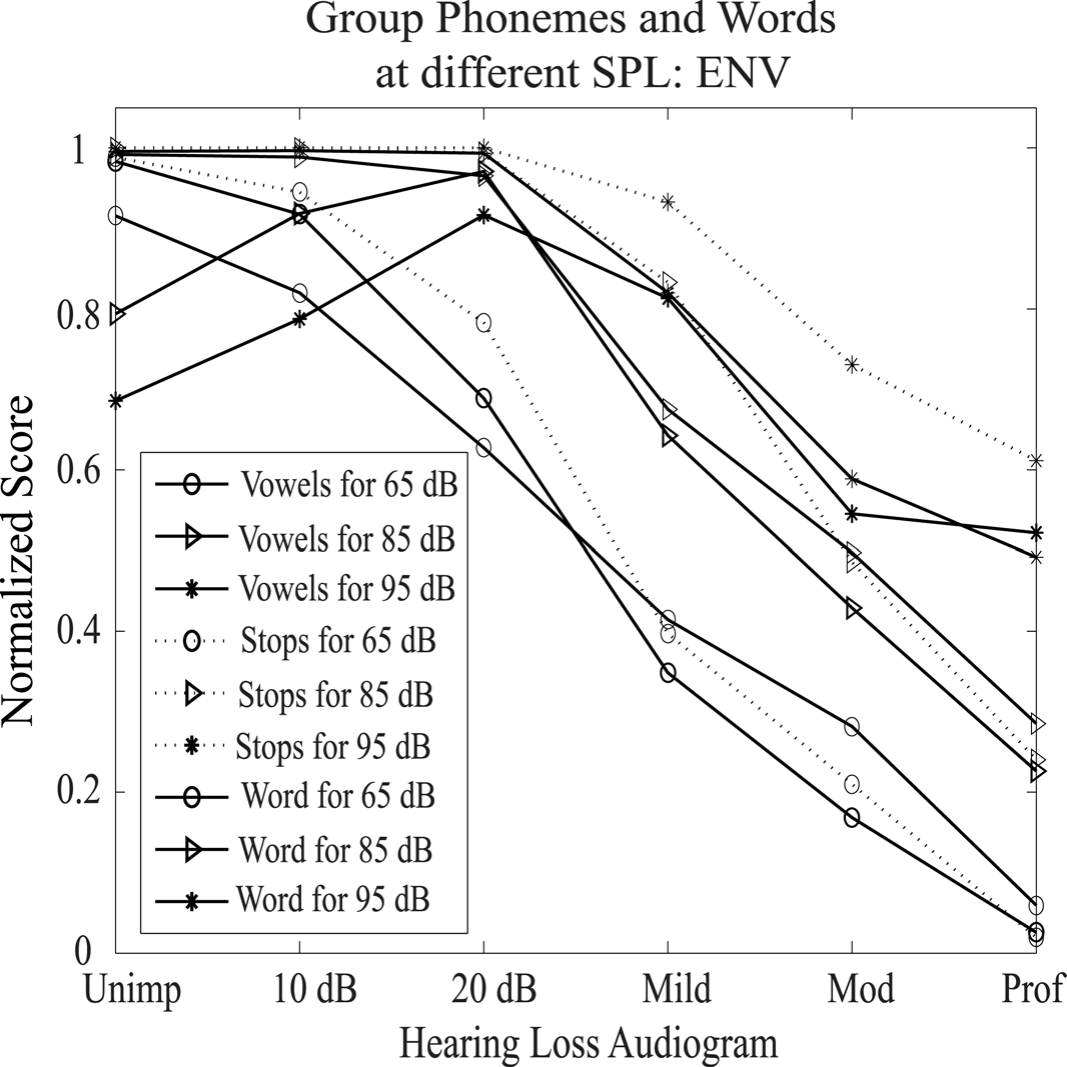
Effects of SPL on the predicted score using the proposed metric. The mean scores for phonemes and words are shown for ENV responses (using *H*_*3*_ feature) at 65, 85, and 95 dB SPL.

In general, speech intelligibility is decreased even for listeners with normal hearing when speech is presented at higher-than-normal levels (above about 80 dB SPL) [50]. Depending on the degree of hearing loss, the decrease in speech understanding is also observed under noisy conditions for people with hearing loss when the speech level is increased beyond conversational speech level [50, 58]. In the literature, this phenomenon is referred to as the roll-over effect. In order to analyze the effects of SPL on the predicted score, this study computed the normalized scores using *H*_*3*_ for two representative phoneme groups (vowels and stops) from TIMIT database and words from NU6 database. The scores were predicted for sound levels at 65, 85, and 95 dB SPL as a function of hearing loss, and the results are shown in Fig 6. It was observed that the estimated speech intelligibility score slightly decreased for words and was almost same for phonemes for listeners with normal hearing as well as for listeners with flat 10 and flat 20 dB hearing loss when speech was presented at higher levels (due to test variability and normative differences, 10–15 dB hearing loss can be treated as normal hearing). This is consistent with the roll-over effect observed for people with normal hearing, indicating that the effects of supra-threshold nonlinearities responsible for this decline was successfully captured by the bispectrum features. However, the predicted score increased substantially for listeners with mild, moderate, and profound hearing loss when speech level was higher than 65 dB SPL. This could be attributed to the increased audibility of the signal (in quiet condition) at higher levels for listeners with hearing loss [59].

### Comparison to the Results from a Behavioral Study

To validate the proposed metric, the subjective speech intelligibility scores from a behavioral study by Studebaker et al. [50] were compared to the scores predicted by the proposed method using the same experimental conditions (for listeners with and without hearing loss both in quiet and under noisy conditions).

#### Speech intelligibility for listeners with hearing loss

**Fig 7:**
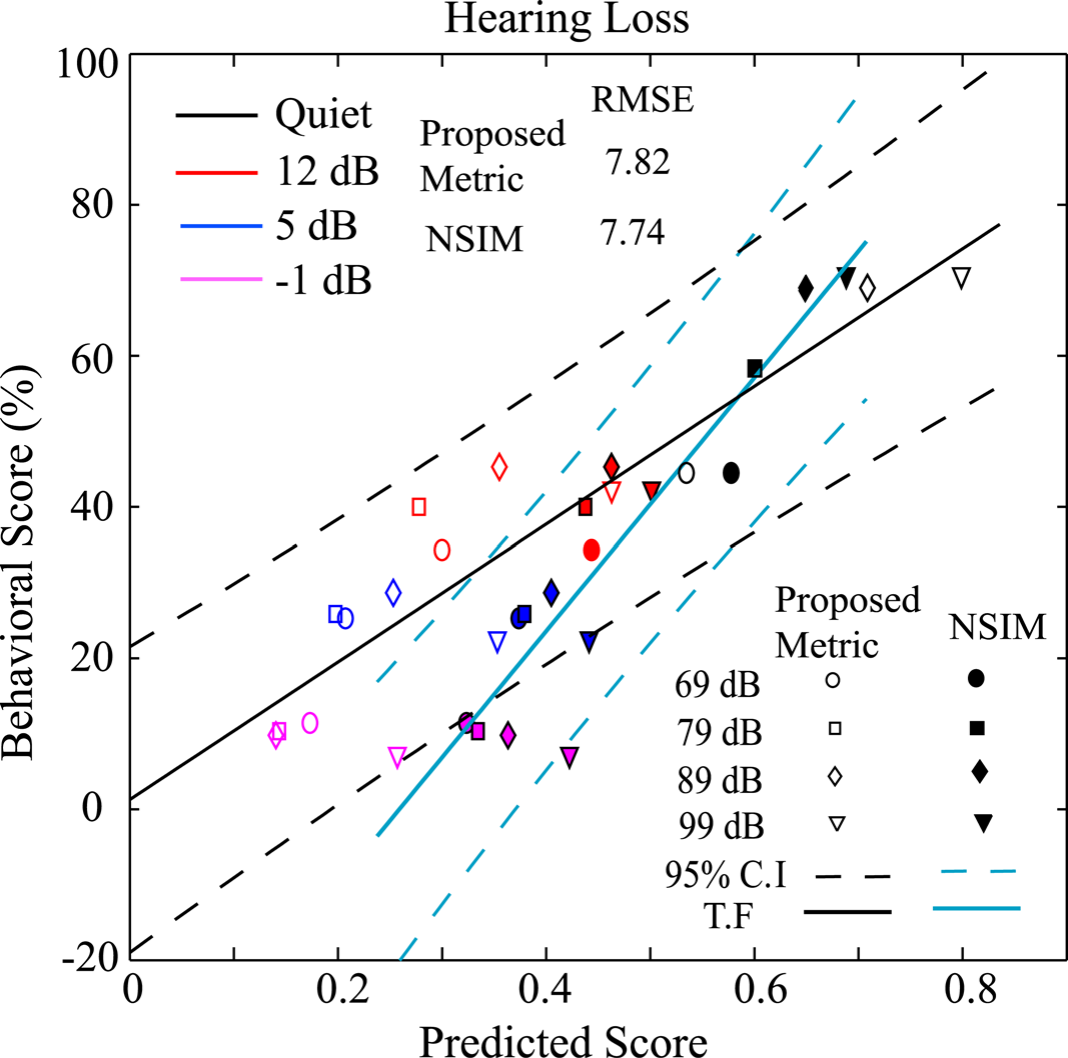
Comparison of subjective scores to the predicted scores for a listener with mild to moderate hearing loss. Responses were simulated in quiet and under noisy conditions using the proposed method (ENV responses using *H*_*3*_ feature) and the full reference NSIM metric. The results are shown for four sound presentation levels (69, 79, 89, and 99) and four SNRs (-1, 5, 12, and in quiet condition). Each point represents the mean score for NU6 words at a particular sound presentation level. The linear regression coefficient between subjective and predicted scores for both the proposed metric and NSIM was found as ~0.92.

Fig 7 shows the word-recognition (NU6 database) scores of a listener with hearing loss (mild to moderate) plotted against predicted scores by the proposed method using ENV responses (open symbols). The performance of a full-reference-based metric, NSIM, has also been shown (filled symbols) for the same conditions. It is to be noted that the acoustic signal property-based methods (e.g., STOI and SII) cannot take into account the effects of hearing loss directly and thus may use an ad-hoc method to predict speech intelligibility scores. For this reason, the scores for listeners with hearing loss were computed using methods based on the responses of the model of the auditory system only.

The audiogram of the hearing-impaired group was described by pure-tone hearing levels of 13, 15, 23, 40, 53, and 52 dB at the octave frequencies of 250, 500, 1000, 2000, 4000, and 8000 Hz, respectively. The parameters of the AN model (C_OHC_ and C_IHC_) were adjusted to match the average hearing loss profile of the listeners who participated in the experiment. Scores were estimated using the proposed method and NSIM for 200 words from the NU6 database in quiet (clean) and under speech-shaped noise at SNRs of -1, 5, and 12 dB with a sound presentation levels of 69, 79, 89, and 99 dB SPL. The mean scores are shown in Fig 7. The relationship between the subjective and predicted (x) scores was fitted by a linear function,

f(x)=a*x+b (6)

where a = 91.17 and b = 1.25 for the proposed metric, and a = 167.56 and b = -43.42 for NSIM.

As shown in the figure, the predicted scores for the proposed metric and NSIM increased as the sound presentation level was increased from 69 to 99 dB SPL in quiet condition, consistent with the recognition scores for the listener with mild to moderate hearing loss from the respective behavioral study. However, the scores for both metrics slightly increased or decreased as a function of SPL under noisy conditions. The dynamic range of subjective scores (~0.03–0.7) was also comparable to that of predicted scores (~0.15–0.8) for the proposed metric. However, the dynamic range of NSIM scores (~0.3–0.7) was lower than the range of subjective scores. Although a function could be applied to extend the dynamic range, a metric with a dynamic range comparable to that of behavioral responses may indicate a plausible strategy employed by the human auditory system to generate those responses.

The linear regression coefficient for both metric was found to be same (~0.92). The root-mean-square error (RMSE) between the behavioral scores and the scores predicted by the fitted function was found to be 7.82 and 7.74 for the proposed metric and NSIM, respectively. The solid line indicates the linear fit, and the dotted lines represent the 95% confidence interval (C.I.) computed for 200 predicted intelligibility scores. All of the data fall within the confidence interval, meaning that the subjective scores can be reliably estimated using the proposed metric.

#### Speech intelligibility for listeners with normal hearing

**Fig 8:**
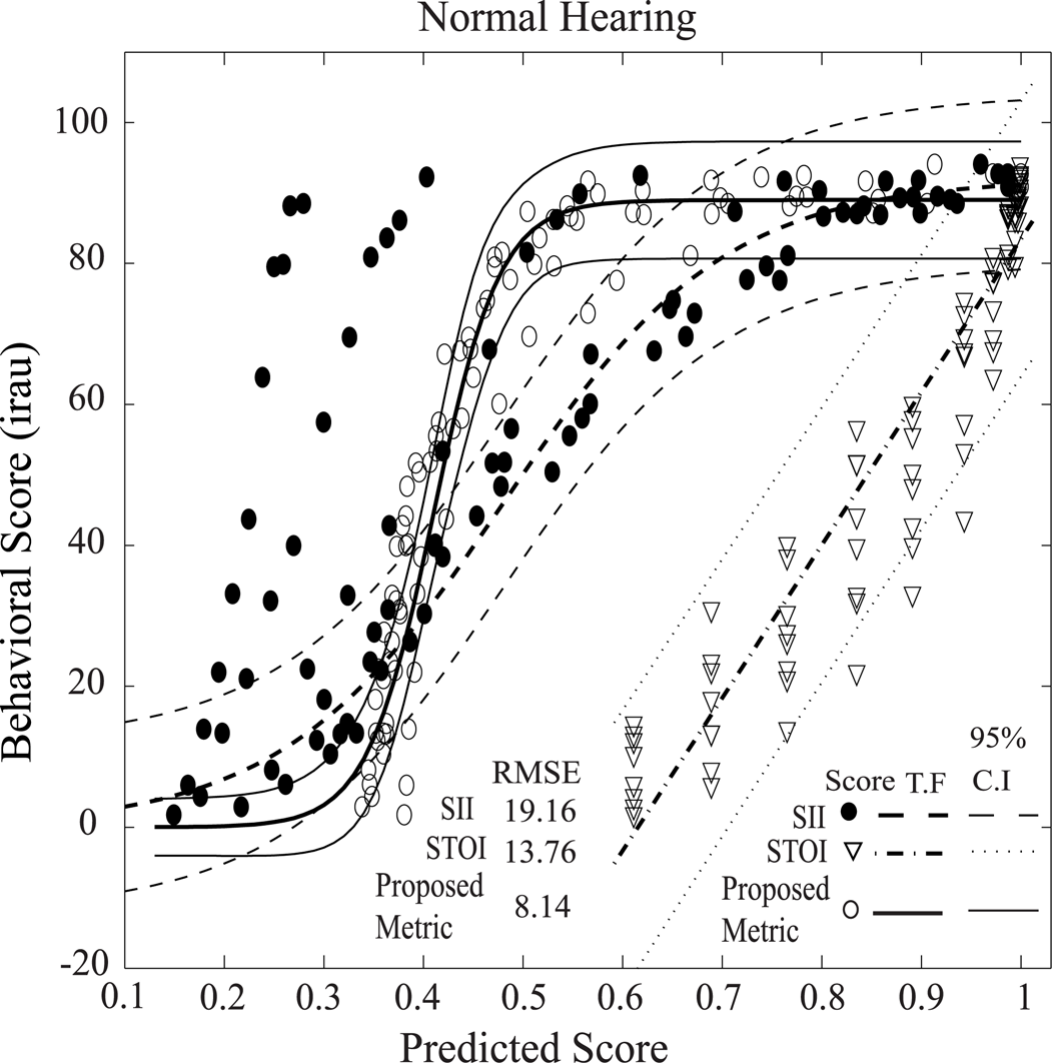
Comparison of subjective scores to the predicted scores for listeners with normal hearing. Three objective measures were considered: the proposed method (ENV responses), SII, and STOI. The scores were estimated and averaged across 200 NU6 words in quiet and under the speech-shaped noise. The results are shown for ten SNRs ranging from -4 to +28 dB and eight different sound pressure levels ranging from 64 to 99 dB SPL. Transfer functions (T. F) with 95% confidence intervals are also shown in the figure for all metric.

Fig 8 shows the relationship between behavioral (subjective) scores from a study by Studebaker et al. [50] and predicted scores for a listener with normal hearing. The scores were predicted using the proposed method as well as two traditional (based on the properties of the acoustic signal) full reference metrics, STOI, and SII [60], for the NU6 words in quiet and under speech-shaped noise at SNRs of -4, -1, 2, 5, 8, 12, 16, 20, 24, and 28 dB for each of the eight speech presentation levels (64, 69, 74, 79, 84, 89, 94, and 99 dB SPL) to match the corresponding behavioral study. In this experiment, the neurograms were constructed by simulating responses of AN fibers for normal hearing, and the scores were estimated using ENV responses (*H*_*3*_) only. The mean subjective and predicted scores were computed across 200 NU6 words for each condition. For SII and the proposed method, the best fit between the subjective and predicted scores was achieved by a sigmoid rather than a linear function, which is described by the following equation:

$$f\left(x\right)=\frac{c}{1+e^{-d\left(m\;*x-k\right)}}$$ (7)

For the proposed method, the parameters were found to be c = 89, d = 3, m = 10, and k = 4.1, and for SII, the best fit was obtained for c = 92, d = 0.9, m = 10, and k = 4.8. However, the best fit for the STOI scores were achieved by a linear equation (as in Eq 6) with the parameters a = 217.06 and b = -133.56. It is to be noted that the current implementation of the STOI did not take into account the effects of sound presentation level on the score, and thus the estimated scores were same at a particular SPL.

In general, the predicted scores progressively declined as the more noise was added to the original signal (in quiet), consistent with the subjective scores observed in the behavioral study. However, the dynamic range of subjective scores (~0–0.94) and SII scores (~0.1–0.96) was substantially higher than the dynamic range of scores for the proposed method (~0.3–1.0) and the STOI (~0.6–1.0). It is clear that the predicted scores using the proposed method fell within the 95% confidence interval of the fitted function, which indicates that the subjective scores could be predicted reliably using the proposed method. However, some of SII scores fell outside the 95% confidence interval of the fitted function. The (RMSE) between the subjective scores and the scores predicted by the fitted functions was found to be 8.14, 19.16, and 13.76 for the proposed method, SII, and STOI, respectively.

## Discussions

In this section, the effects of the degree of hearing loss, noise, audibility, and sound presentation level on the bispectrum (i.e., phase coupling) are presented in order to illustrate how the proposed metric works. The distribution of predicted scores across phonemes and subjects is also described.

### Effects of Hearing Loss on Phase Coupling

**Fig 9:**
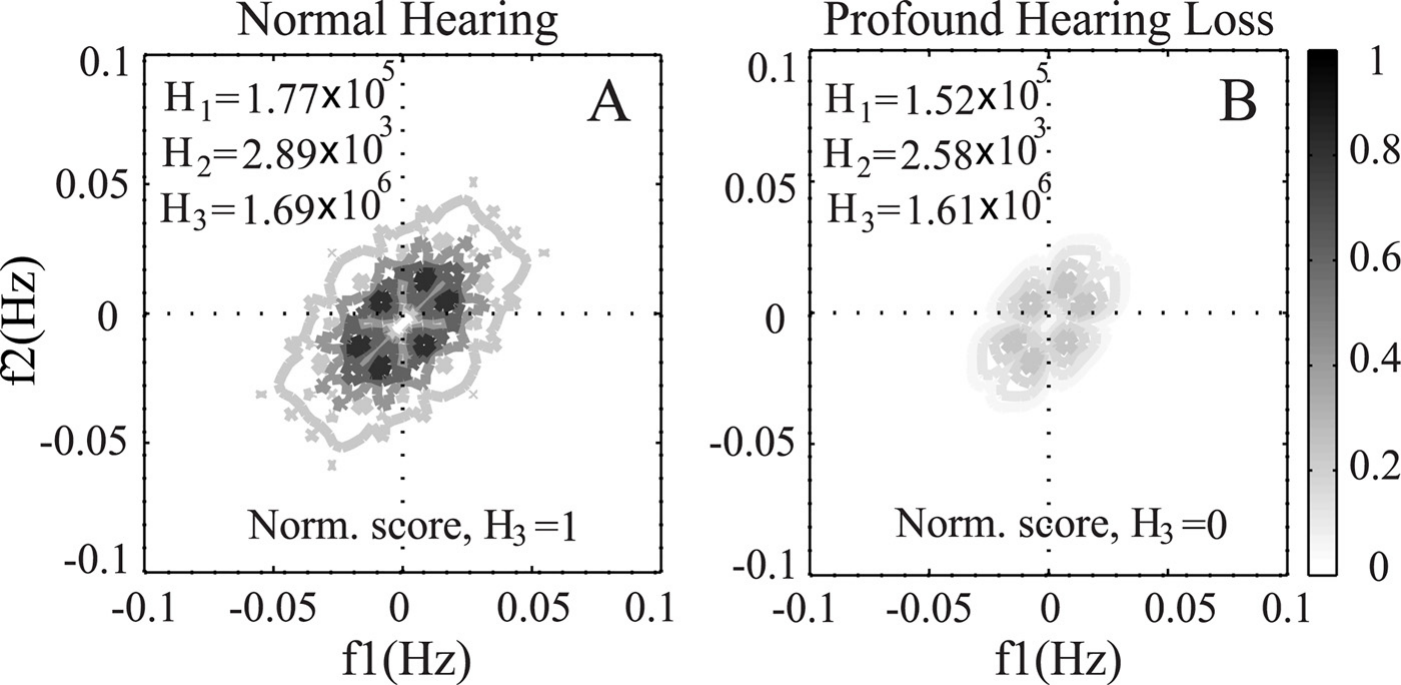
Illustration of the effect of hearing loss on bispectrum. The bispectrum contour map was derived from the ENV responses for listeners with normal hearing (A), and profound hearing loss (B). The range of frequencies over which phase coupling was observed and also the maximum magnitude changed as a function of hearing loss.

As mentioned earlier, the bispectrum is a method to detect the presence of quadratic phase coupling among different frequency bands in a signal. To study how the distribution of phase coupling of a clean signal changes as a function of the degree of hearing loss, the contour maps of the bispectrum for a typical speech word (such as /use/ taken from NU6) are shown in Fig 9. The word was presented at a 65 dB SPL.

The bispectrum was calculated after simulating the ENV responses of the AN model as a function of hearing loss. The bispectrum contour maps for listeners with normal hearing and profound hearing loss are shown in the panels A and B, respectively. The presence of phase coupling was indicated by the peaks in the bispectrum of the signal. It is clear that the extent (range of frequencies) and magnitude of phase coupling degraded substantially in the case of profound hearing loss as the signal is inaudible in most frequencies. For other intermediate profile of hearing loss, the feature values fell within these two extreme cases (Fig 5). Thus the features extracted from the bispectrum were able to estimate speech intelligibility scores for people with different degree of hearing loss.

### Effects of Noise on Phase Coupling

**Fig 10:**
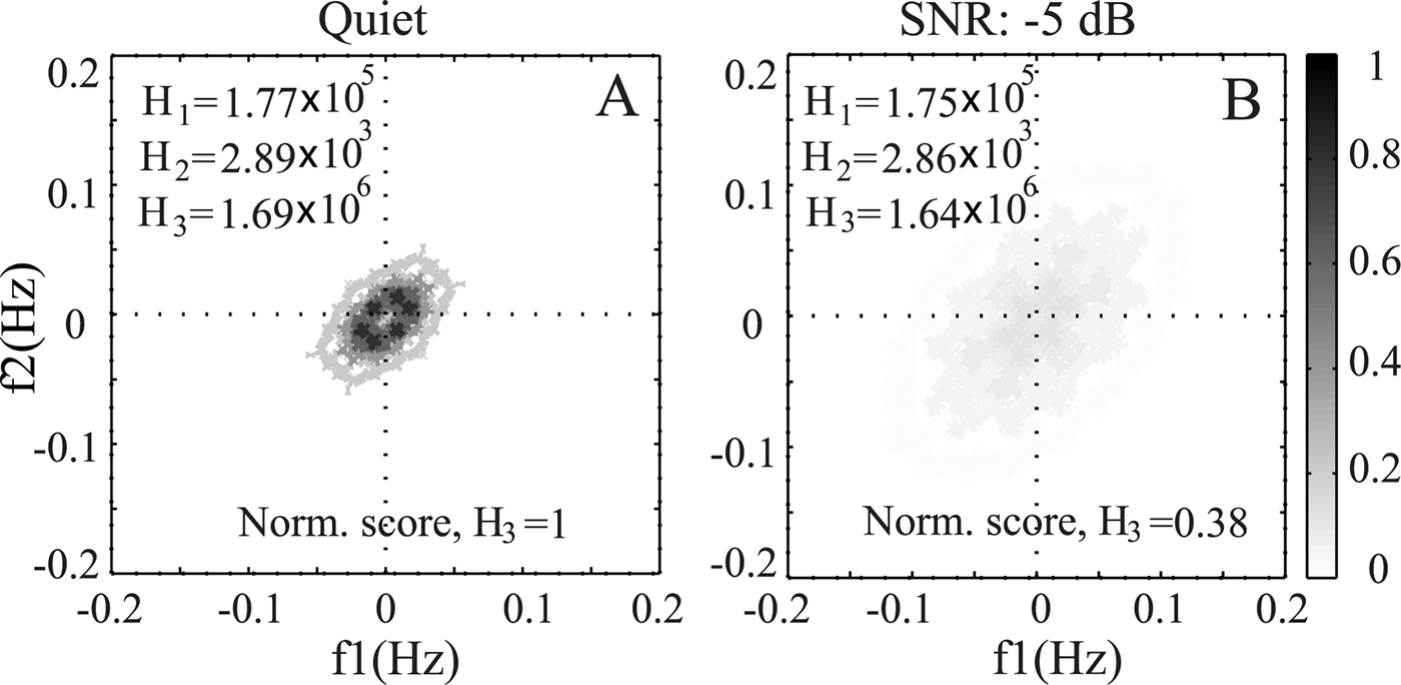
Illustration of the effect of noise on the bispectrum. The bispectrum contour maps were estimated from the ENV neurogram responses (for a listener with normal hearing) to a typical word /use/: (A) in quiet, and (B) under speech-shaped noise at an SNR of -5 dB. The presentation level of the signal was at 65 dB SPL.

Fig 10 shows the bispectrum contour map from the ENV neurogram responses (for listeners with normal hearing) of a typical speech word (/use/ taken from NU6 database) presented at a 65 dB SPL in quiet (panel A) and under noisy condition at an SNR of -5 dB (panel B). The peaks in the bispectrum represent the phase coupling among frequency components. It is obvious that the phase coupling in quiet condition occurred to a smaller frequency range with relatively higher magnitudes. However, when noise was added to the original signal, the magnitude of phase coupling decreased substantially, and the frequency range over which phase coupling was observed increased (in Fig 10B). Thus the predicted score changed systematically as a function of SNR, and the results were described in Fig 8.

**Fig 11:**
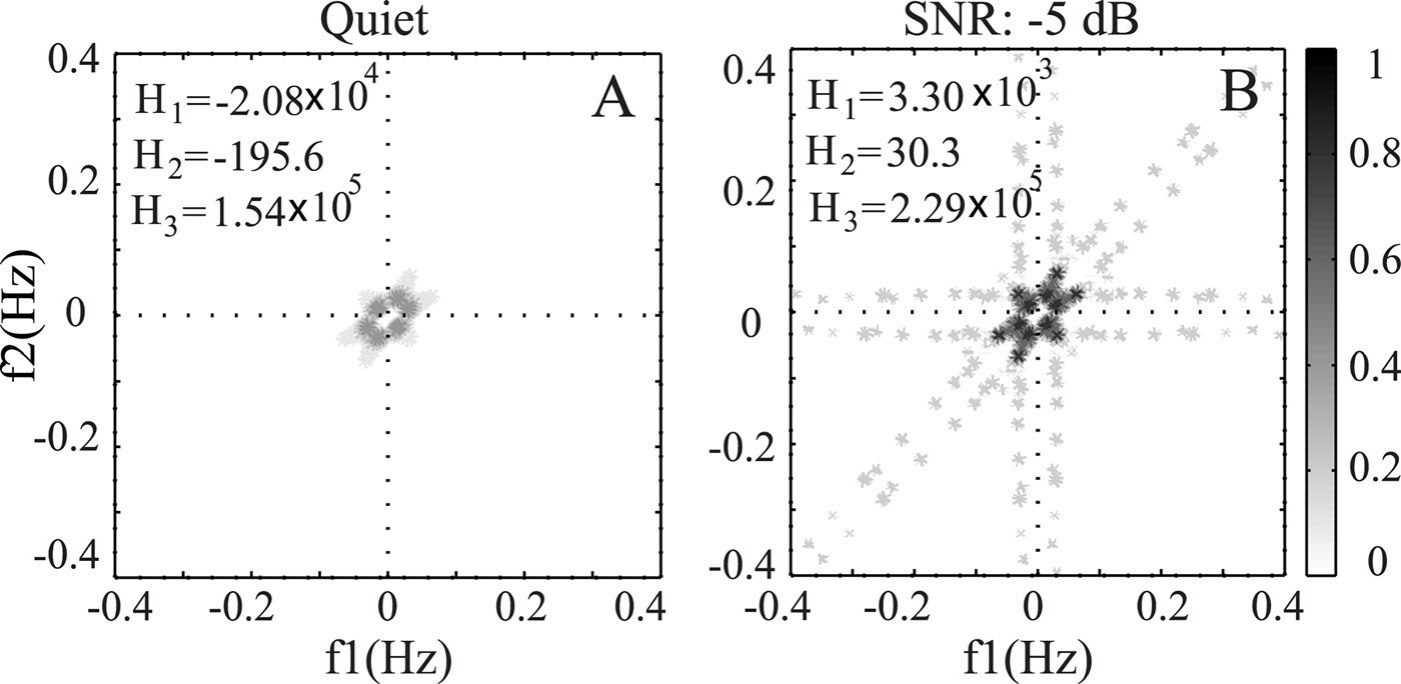
Contour maps of bispectrum for a speech word (/use/): (A) for clean signal, and (B) for noisy signal (additive white Gaussian noise) with an SNR of -5 dB.

In order to illustrate the effect of noise on phase coupling for acoustic signal in the time domain (i.e. without using AN model responses), the bispectrum was calculated for the same word (/use/) under clean and noisy conditions (as used in Fig 10), and the contour plots are shown in Fig 11. The same technique and parameters were used to estimate the bispectrum coefficients for the acoustic signal. As shown in the figure, the frequency range and magnitude of phase coupling increased when noise was added to the clean signal, in contrast to the pattern observed for the neural responses (in Fig 10). This was due to the fact that neurogram responses were constructed from the responses of a bank of band-pass (i.e., basilar membrane) filters, and thus only the interaction among the frequency components within the filter was considered, whereas the interaction among all frequency components was captured in the bispectrum for the signal in the time domain. Again, the clean signal showed a restricted region of phase coupling, whereas the noisy signal showed phase coupling over a larger range of frequency components.

### Effects of SPL on the Bispectrum

**Fig 12:**
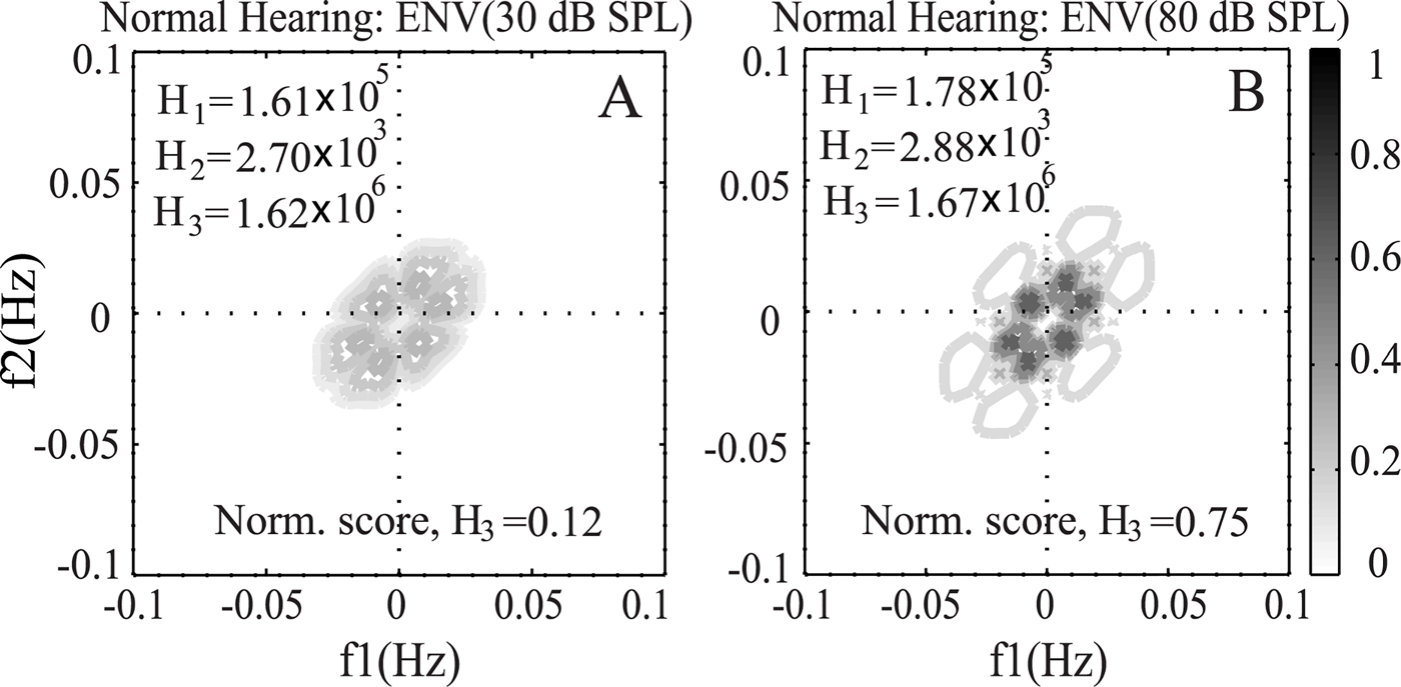
Effects of SPL on the bispectrum. Results are shown for a listener with normal hearing in response to a speech signal (word: /use/) at 30 and 80 dB SPL. A: Bispectrum at 30 dB SPL using ENV responses. B: Bispectrum at 80 dB SPL using ENV responses.

In order to illustrate the effect of speech presentation level on the features of the bispectrum, Fig 12 shows the bispectrum calculated from the ENV responses to a typical speech word (/use/) taken from NU6 database. The results are shown for two different speech presentation levels such as 30 and 80 dB SPL. It is clear that the magnitudes of the bispectrum peaks for 80 dB SPL (panel B) are higher than the magnitudes of the bispectrum for 30 dB SPL (panel A). Also, the extent of frequency over which phase coupling was observed was substantially wider for the responses at higher SPL compared to the range of frequencies observed in the bispectrum of responses at lower SPL. This was due to the fact that the auditory filter becomes broader at higher SPL than the bandwidth of the filter at lower SPLs. The more frequency components passed into the filter for higher SPL, and thus more phase coupling occurred. However, the magnitude of phase coupling was not a simple linear function of audibility (compare Figs 9A–12). Fig 9A shows the contour map of bispectrum at 65 dB SPL for exactly the same conditions (except SPLs) for which the responses are shown in Fig 12, and it is obvious that the highest magnitude of phase coupling occurred at 65 dB SPL (conversational speech level) compared to the responses at a lower and higher SPLs. This behavior is consistent with the roll-over effect observed even for the listeners with normal hearing at higher-than-normal levels [50]. This indicates that the effects of auditory supra-threshold nonlinearities were also reflected in the bispectrum, which has also been discussed in the following section. The effect of SPL on the predicted score was shown and discussed in Fig 6.

### Effects of Audibility on Phase Coupling

**Fig 13:**
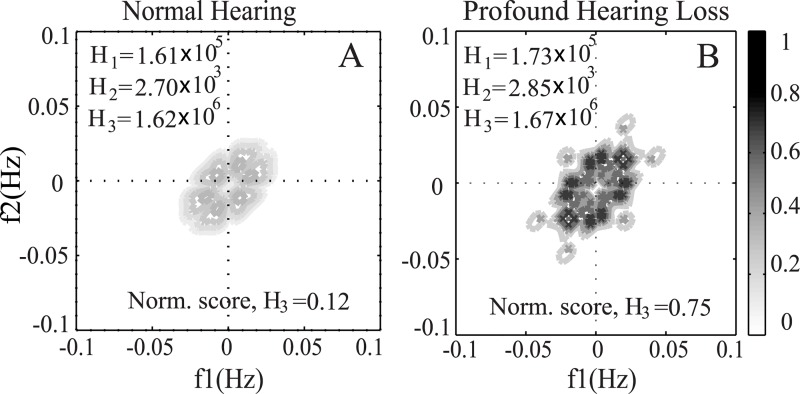
Effects of audibility on the bispectrum. Results are presented for ENV responses of a typical speech word (such as /use/). A: Bispectrum contour map for a listener with normal hearing at 30 dB SPL. B: Bispectrum contour map for a listener with flat 70 dB hearing loss at 100 dB SPL.

Fig 13 shows the contour map of bispectrum calculated from the ENV responses of a typical speech (word: /use/ taken from NU6 database) to illustrate the effects of audibility on phase coupling of neurogram bispectrum. The left panel (A) represents the bispectrum contour map for a listener with normal hearing at 30 dB SPL, and the right panel (B) shows the contour map for a listener with flat 70 dB hearing loss (moderate to profound) at 100 dB SPL.

The audibility of the signal at all frequencies for both cases was maintained at 30 dB above threshold. It is obvious that the magnitude of phase coupling and the range of frequencies over which phase coupling occurred were substantially higher and wider, respectively, for the listener with flat 70 dB hearing loss compared to those of a listener with normal hearing. The effect (presence or deficit) of supra-threshold nonlinearities incorporated in the AN model was successfully captured by the bispectrum features. This indicates that the predicted scores for the listener with hearing loss not only reflect the effect of audibility of the signal but also takes into account the effect of nonlinearities at higher presentation levels. It is to be noted that the magnitude of features at 100 dB SPL for the listener with flat 70 dB hearing loss was lower than the respective features at 65 dB SPL for a listener with normal hearing (Fig 9A), and thus the predicted score for the listener with hearing loss never reached near 100% irrespective of the sound presentation levels (Fig 7). This observation is also consistent with the behavioral responses (for the similar condition) reported in the literature [50].

### Distribution of Predicted Scores across Subjects and Phonemes

Typically, there is more variability in behavioral responses across subjects with hearing loss than for subjects with normal hearing [61, 62]. However, the variability in recognition scores could also be resulted from the test itself (e.g., test materials, test-forms, and test scores) [63]. Recently, few studies show that noise exposure and aging can cause a loss of significant proportion of AN fibers without any substantial effect in detection thresholds [64, 65]. Moreover, this kind of neuropathy appears to preferentially affect lower spontaneous rate (SR) AN fibers [66, 67]. In order to explore the variability in recognition scores across subjects with similar audiograms, the scores were predicted using the proposed method by considering the neural responses from only high-SR fibers (i.e., cochlear neuropathy) as well as from all of the three types (low, medium, and high) of SR fibers. Similarly, responses from a range of subjects (with similar audiogram, but with different degree of cochlear neuropathy) can be simulated by applying a range of weights to different types of AN fiber responses.

**Fig 14:**
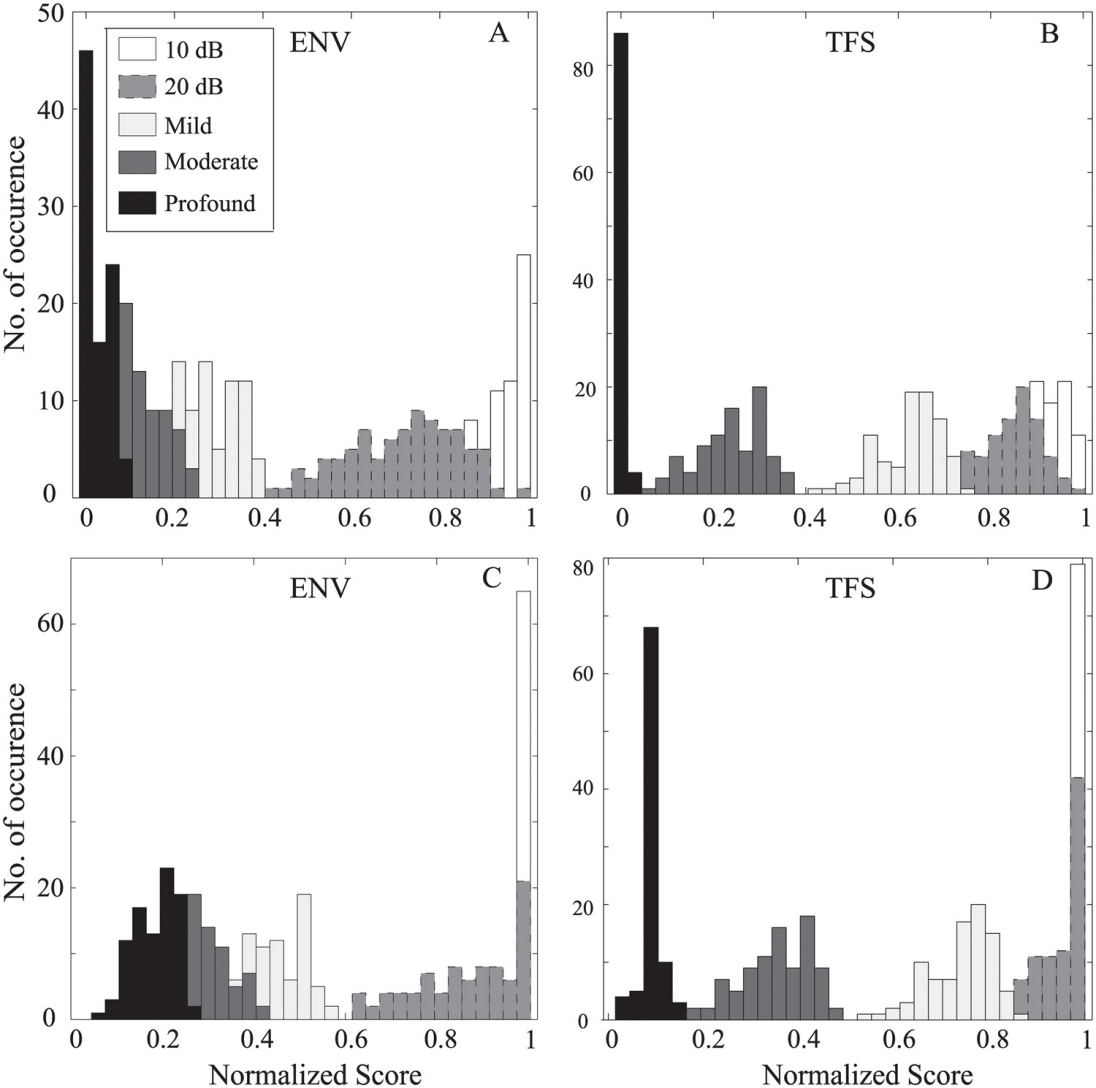
Distribution of speech intelligibility scores. Scores were estimated using the proposed method for affricate phonemes with five types of hearing loss profiles. (A, B): Using ENV and TFS responses of three types (high, medium, and low spontaneous rates) of AN fibers; (C, D): Using ENV and TFS responses of only high SR AN fibers.

Fig 14 shows the histogram of predicted speech intelligibility scores for listeners with five different hearing loss profiles. The scores were estimated using the proposed method in response to affricates phonemes extracted from the TIMIT database. The distribution of scores for ENV and TFS responses using three types of SR AN fibers is presented in the upper panels (A, B), and the lower panels (C, D) show the distribution of scores using responses from only high SR fibers. The scores for both cases were well-separated as a function of hearing loss. However, the variability in the predicted scores observed in each panel for a particular hearing loss profile represents the distribution of scores across phonemes for the same listener, whereas for the same profile of hearing loss, the difference between panels A and C (and also B and D) indicates the variation across subjects. The scores for listeners with a flat 10 dB hearing loss were very close and comparable to the scores from listeners with normal hearing. In general, it was observed that the magnitude of predicted speech intelligibility scores for listeners with cochlear neuropathy shifted towards slightly higher values compared to the predicted scores using all SR fiber responses. It is expected that predicted scores for other subjects with similar audiograms (but with different degree of cochlear neuropathy) will fall in between the ranges reported in panels A and C for ENV responses (and panels B and D for TFS responses).

### Limitations of the proposed metric and potential future works

In general, it is assumed that any objective speech intelligibility measure having the same dynamic range and a linear relationship with the subjective scores would indicate a plausible strategy employed by the human auditory system to produce the behavioral responses. In this regard, it is obvious that although the proposed metric had a linear relationship with the subjective scores for listeners with hearing loss (Fig 7), the best fit between the subjective and predicted scores was obtained by a sigmoid function for a listener with normal hearing (Fig 8). Few possibilities might contribute to the shortcomings of the proposed metric. The AN model used in this study did not capture all of the nonlinearities available at the level of the auditory periphery, i.e., the model is not perfect. Also, a more accurate model of the central auditory system is needed. In this study, extracting bispectral features from the auditory neurogram was assumed as a central mechanism to predict recognition scores; however, the physiological correlate is not obvious from the literature.

Perceptual studies of speech generally use human subjects, and thus a specialized neural mechanism is thought to be responsible for speech perception. However, the AN model used in this study was largely based on the physiological data from animal studies, especially from cat. It is well-known that physiological properties of many neurons show strikingly similar properties in unrelated species such as rodents, cats, and bats, suggesting a more general auditory neural mechanism common to mammals including human. Thus, it is likely that although the predicted scores are based on the simulated neural responses for nonhuman species, a great deal can be learned about the neural processing of speech by human auditory system.

In order to model each audiogram, the values of two parameters, C_OHC_ and C_IHC_, were chosen such that approximately two-thirds of the threshold shift measured in the audiogram can be attributed to OHC impairment and one-third to the IHC impairment. However, there is substantial variability across individuals [52]. As a future work, a number of different patterns of pathology that would be consistent with the audiogram could be explored systematically in order to account for the variability observed across listeners (from behavioral studies). For example, a different combination of OHC and IHC impairment could be applied to produce the same audiogram (i.e., the parameters will not be unique for a given threshold shift), and thus the proposed metric would allow analyzing the effects of different pathology on estimated scores. In addition, the pathology could also be modeled by a combination of different weights for the responses of different SR AN fibers. In this study, only two extreme conditions (all fibers vs. only high SR fibers) were considered (Fig 14). Overall, it is a great advantage of the proposed metric over other traditional methods (which can show only the averaged behavior) that this computational-model-based method can deal with the variability in the scores across listeners as well as the input speech materials.

In the literature, the subjective speech intelligibility score is measured using phonemes, words, or sentences [68]. However, the proposed metric was successful in predicting speech intelligibility for phonemes and words only. The bispectral features for both phonemes and words were relatively stable for two extreme conditions (Fig 4), whereas the H_1_, H_2_ and H_3_ estimated from the neurogram of sentences varied substantially depending on the length of the sentences (results not shown). Thus the proposed reference-free metric could not be extended to predict intelligibility for sentences.

## Conclusions

This study proposed a reference-free metric to predict speech intelligibility for people with hearing loss and also for listeners with normal hearing in quiet and under noisy conditions. For this, the responses of AN fibers were simulated to construct neurograms, and then bispectrum features were computed to estimate speech intelligibility. In this study, three features from the bispectrum were identified whose magnitude was relatively constant (irrespective of input speech signal) for a particular profile of hearing loss, and these features consistently changed as a function of signal-to-noise ratio. Thus unlike other full reference-based metrics, the responses to clean signals were not required to predict speech intelligibility using the proposed method. The presence or deficit of supra-threshold nonlinearities captured in the neural responses for listeners with normal hearing or hearing loss was also reflected in the phase coupling information of the neurogram bispectrum. Although the dynamic range of feature values for TFS responses was larger compared to the feature values from ENV responses, the normalized score using ENV responses showed a relatively more gradual declination with hearing loss. The performance of the proposed metric was compared to the subjective scores from a behavioral study, and the results showed a good fit with a small error suggesting that the subjective scores can be estimated reliably using the proposed neural-response-based metric.
